# A common, non-optimal phenotypic endpoint in experimental adaptations of bacteriophage lysis time

**DOI:** 10.1186/1471-2148-12-37

**Published:** 2012-03-19

**Authors:** Lynne Chantranupong, Richard H Heineman

**Affiliations:** 1 Department of Biology, Massachusetts Institute of Technology, Cambridge, MA, 02139, USA; 2 Department of Chemical Engineering, University of Texas at Austin, Austin, Texas 78712, USA; 3 Institute for Molecular Virology, University of Minnesota, 18-242 Moos Tower 515 Delaware St. SE, Minneapolis, MN 55455, USA; 4 Section of Integrative Biology, University of Texas at Austin, Austin, Texas 78712, USA

**Keywords:** Experimental evolution, ΦX174, Optimality, Life history evolution, Genetic constraint, Bacteriophage, Lysis, Molecular evolution, Virulence, Phenotype prediction

## Abstract

**Background:**

Optimality models of evolution, which ignore genetic details and focus on natural selection, are widely used but sometimes criticized as oversimplifications. Their utility for quantitatively predicting phenotypic evolution can be tested experimentally. One such model predicts optimal bacteriophage lysis interval, how long a virus should produce progeny before lysing its host bacterium to release them. The genetic basis of this life history trait is well studied in many easily propagated phages, making it possible to test the model across a variety of environments and taxa.

**Results:**

We adapted two related small single-stranded DNA phages, ΦX174 and ST-1, to various conditions. The model predicted the evolution of the lysis interval in response to host density and other environmental factors. In all cases the initial phages lysed later than predicted. The ΦX174 lysis interval did not evolve detectably when the phage was adapted to normal hosts, indicating complete failure of optimality predictions. ΦX174 grown on slyD-defective hosts which initially entirely prevented lysis readily recovered to a lysis interval similar to that attained on normal hosts. Finally, the lysis interval still evolved to the same endpoint when the environment was altered to delay optimal lysis interval. ST-1 lysis interval evolved to be ~2 min shorter, qualitatively in accord with predictions. However, there were no changes in the single known lysis gene. Part of ST-1's total lysis time evolution consisted of an earlier start to progeny production, an unpredicted phenotypic response outside the boundaries of the optimality model.

**Conclusions:**

The consistent failure of the optimality model suggests that constraint and genetic details affect quantitative and even qualitative success of optimality predictions. Several features of ST-1 adaptation show that lysis time is best understood as an output of multiple traits, rather than in isolation.

## Background

Models that describe evolutionary processes must strike a balance between simplicity and detail. At one end of the spectrum are optimality models, which predict phenotypic evolution based on natural selection in the context of ecology, often neglecting genetics except in the form of simple 'trade-offs' between phenotypic alternatives. The appeal of such models is in their apparent generality, enabling prediction across a wide variety of organisms and environments with minimal input about genetic parameters. The weakness of optimality models is argued to lie in their neglect of genetic details, the very matrix on which the evolutionary process rests [1]. If these details lead to constraint or unexpected pleiotropic interactions between phenotypes, optimality model predictions may mislead.

There is a long history of disagreement about the importance of genetic details in understanding phenotypic evolution [2]. There seems to be little resolution. Optimality models are still widely applied with apparent success for some biological systems [3-10], but studies that account for genetic details seem to gain insight from their incorporation [11-14]. As yet, however, there are few studies that both enlist information about genetic details and conduct quantitative tests of optimality model predictions. Such direct comparisons of the two approaches are essential in resolving the merits of genetic details in understanding evolution.

Here we use experimental evolution of bacteriophage to test an optimality model for a life history trait, lysis time. Environmental conditions are easily controlled when growing bacteriophages, making it simple to parameterize and test quantitative predictions about optimality in reasonably well-understood genetic systems. The lysis time model has been tested on three phages, λ, T7, and T4 [15-17], but the present work extends these tests to a pair of phages with a very different lysis mechanism.

### Lysis time optimality model

**Figure 1:**
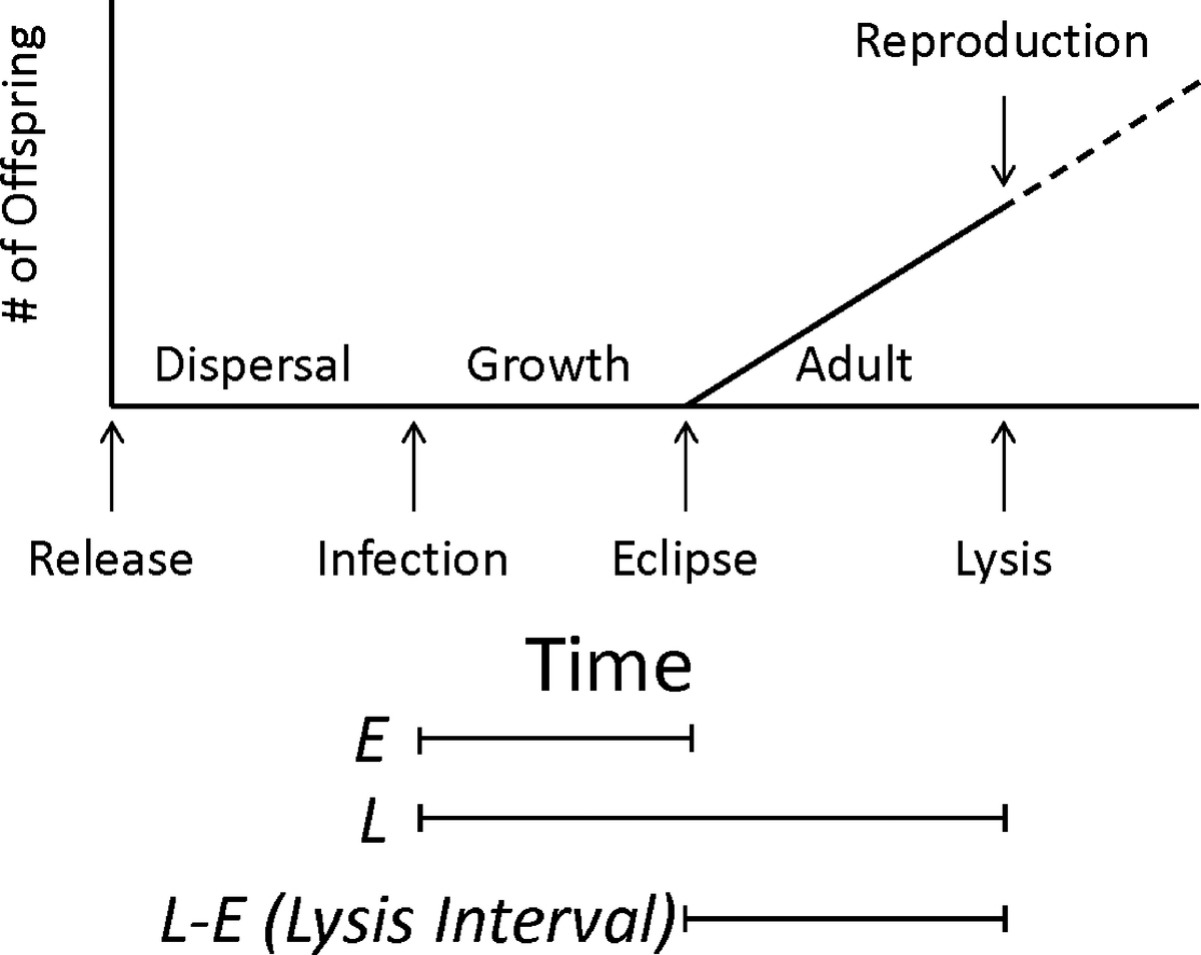
**Lytic bacteriophage life cycle**. Lytic bacteriophages are viruses of bacteria with a life cycle similar to that of other organisms that breed only once, such as the agave plant or salmon. There are three main stages, analogous to the dispersal, growth, and reproductive periods in animals and plants. (1) Dispersal stage. The virus floats freely in the environment until it encounters a host, after which it has a chance to infect the host that is determined by the phage's adsorption constant. (2) Growth stage. The phage genome enters the cell and turns the infected host into a phage factory by creating viral components. During this period the initial phage virion is destroyed but no new ones have been generated. The end of this period, as the first viable progeny is created, is the eclipse time (*E*). (3) Adult stage. The rate of phage accumulation within the cell is approximately linear across diverse phage lineages [15,18,19], although in some cases, such as phage T3 and T7, production seems unable to continue indefinitely [20,21]. This third stage continues until lysis (*L*), in which the phage bursts its host cell in order to escape it and infect new hosts. The period from first phage production to lysis is here called lysis interval. Lysis terminates phage production and initiates a new dispersal stage. Figure modified from [17].

Most bacteriophages lyse their hosts to enable progeny to escape into the environment (Figure 1). Lysis of the infected cell releases all phage progeny at once and so is the phage equivalent of the age of reproduction. Furthermore, this life history trait has major effects on phage fitness. The infected cell typically produces phages indefinitely until lysis, so a long lysis interval increases progeny but extends generation time, and vice versa.

A simple optimality model predicts which lysis interval will maximize fitness simply as a function of the intrinsic growth rate of the phage population [22,23]. The optimality model assumes linear accumulation of phages within the cell and parallels a theory of optimal foraging in patchy environments originally developed by Charnov [24]. It leads to an approximate lysis time optimum of

(1) $$\hat{L}-E=\frac{1}{\hat{r}}$$

[16,23]. The optimal lysis time ($\hat{L}$) minus the eclipse time (*E *, when functional phage progeny begin to accumulate inside the cell) should evolve to equal the inverse of *r *(the intrinsic rate of increase, a measure of growth rate). $\hat{L}-E$ is defined here as the lysis interval. All viable phage progeny are produced inside the cell during this interval, at an approximately constant rate (Figure 1). Since this equation applies at the evolutionary equilibrium, the optimal lysis interval changes when the environment changes or the phage evolves in response to a change in the environment.

The simple model presented here ignores the effects of non-genetic lysis variation. Lysis asynchrony (in which genetically identical phages lyse at different times due to phenotypic noise) is important in ΦX174 -like phages and affects the optimal lysis interval. A more complete model that accounts for this parameter was used for optimality predictions throughout (see equations 2 and 3 in Methods for details).

This model can be explored with experimental evolution [15-17,25]. As the genetic basis of lysis is well studied and apparently simple for many phages, lysis time is an ideal system for exploring how optimality interacts with genetic details in a life history trait.

### Lysis in isometric phages

Only two assumptions about lysis mechanisms are required by the optimality model; both are met in ΦX174 bacteriophage. First, average lysis time is evolvable, i.e. mutations exist that can change it [18,26]. Second, phage progeny accumulate linearly within the infected cell until the end of the lysis interval [18,27]. By similarity of genome organizations, we assume ST-1 also meets these assumptions.

ST-1 and ΦX174 are isometric, icosahedral phages of the family microviridae with small single-stranded DNA genomes. ΦX174 has 11 genes, while ST-1 encodes homologs to those 11 plus an extra region with putative genes, like certain other isometric phages [28]. In both phages, only one gene (gene *E*) is directly implicated in lysis [29,30], and it overlaps, out of frame, the highly conserved gene *D *[28] which encodes the external scaffolding protein [31].

The gene *E *protein, GpE, causes lysis by interfering with cell wall synthesis during cell division [32,33], a simpler mechanism than that seen in larger phages [34]. All mutations in gene *E *known to affect lysis do so by altering expression or by eliminating lytic activity altogether [27,35]. GpE noncompetitively inhibits MraY [36], and without this enzyme the cell expands and eventually lyses rather than dividing [37].

ΦX174 requires the host factor slyD for lysis [29,38,39]. Mutations in gene *E *that increase gpE expression can restore lysis [35,40]. In addition, when these mutant alleles were expressed from a λ phage construct they hastened mean lysis time in the presence of slyD [26], suggesting a potential for quantitative variation in ΦX174 lysis time.

We adapted these two phages across serial flask transfers for hundreds of generations to identify the mechanisms and generality of evolution to an optimal lysis time. The universal outcome for ΦX174 adaptations was a lysis interval indistinguishable from that of wild-type, despite the predictions of the optimality model that a shorter lysis interval should evolve. In ST-1, the lysis interval shortened somewhat but did not reach the predicted optimum.

## Results

We adapted phages under a variety of environmental conditions to determine if they evolved to lyse at the predicted optimal time. In every replicate, the lysis interval remained longer than the optimality model predicted. Changes in the single known lysis protein occurred only when phages were adapted on hosts that eliminated wild-type phage lysis entirely.

Lysis time is the sum of eclipse and lysis interval (before and after phage progeny start to accumulate in the cell, respectively) and the model assumes that eclipse time is fixed and that the optimal lysis time is achieved by changes in the lysis interval. We thus address primarily the lysis interval.

### ΦX174 phenotypic adaptation to *E. coli *C: Failure to evolve shorter lysis time

We adapted ΦX174 to *E. coli *C twice under similar conditions, in which the predicted optimal lysis interval was shorter than the initial lysis interval. Thus we expected the lysis interval to shorten over adaptation. One adaptation started with wild-type phage, while the other started with a recombinant population to increase initial variation. Through this latter mechanism, ΦX174^+ ^was provided with substitutions from two lines preadapted to conditions differing slightly from those used here. Higher genetic variance in the population should facilitate adaptation. Preliminary analysis showed that total lysis time was broadly similar between the two adaptations, so an isolate from the recombinant population (ΦX174_C_) was chosen for further characterization.

Following 34 hours of adaptation, ΦX174_C _fitness increased from 20.8 to 23.4 db/hr, an increase of 2.6 db/hr (Table 1, *p *< 0.03 by 1-tailed *t*-test). No other phenotypes measured changed significantly, although eclipse time was possibly shorter by ~1 min (*p *< 0.07 by 2-tailed *t*-test, Table 1). The lysis interval (11.4 min) did not evolve to the predicted optimum of 5.5 min (calculated using lysis time variance as in equation 3, *p *< 0.0002 by the parametric model).

**Table 1:** Phenotypic traits of phage lines with standard errors (computed from observations) and number of assays

	ST-1^+^	ST-1_K-12_	ΦX174^+^	ΦX174_C_	ΦX174_sly D_	ΦX174_sly D _C cells	ΦX174_sly D low_
Optimal lysis interval*	4.4	4.7^£^	5.2	5.5	5.5		8.7
Lysis interval*	10.4 ± 0.20 (2)	8 ± 0.13 (13)	8 ± 0.89 (2)	11.4 ± 1.2 (3)	11.2 ± 0.54 (4)	8.9 ± 0.44 (3)	10 ± 0.11 (2)
Lysis interval variance^†^	9.2 ± 2.1 (2)	1.3 ± 0.19 (3)	6.4 ± 3.5 (2)	13.2 ± 6.0 (3)	9.3 ± 1.7 (4)	9.6 ± 2.8 (3)	4.3 ± 12.2 (2)
Eclipse time*	7.7 ± 0.013 (2)	5.8 ± 0.05 (3)	7.7 ± 0.11 (2)	6.5 ± 0.015 (3)	9.2 ± 1.4 (4)	8.9 ± 0.50 (2)	8.8 ± 0.05 (2)
Fitness^‡^	30.4 ± 1.1 (3)	19.5 ± 1.0 (5)	20.8 ± 0.64 (3)	23.4 ± 0.05 (3)	21.2 ± 0.3 (3)	20.4 ± 0.59 (3)	10.6 ± 0.37 (4)
Burst size	222 ± 35 (9)	160 ± 24 (8)	267 ± 8 (2)	283 ± 33 (3)	148 ± 34 (2)		
Adsorption^§^	8.3 ± 0.61 (3)	8 ± 0.66 (3)	7.2 ± 0.66 (3)	7.7 ± 0.97 (3)	5.9 ± 2.0 (3)		

*minutes, ^†^minutes^2^, ^‡^doublings/hour, ^§^10^-9 ^ml/min

^£^Under phase II conditions; under phase I conditions the optimal lysis interval is 2.7 min and fitness is 37.3 db/hr

The failure of the mean lysis interval to adapt closer to the optimum over ~120 generations means that even qualitative predictions of evolution failed for ΦX174_C_. Within the framework of our model, selection for earlier lysis did exist; from projections based on measurements of life history traits, the evolved phage was 4.3 db/hr away from the fitness it might have reached at optimality. This potential fitness advantage is large compared to the fitness difference of 2.6 db/hr between the initial and evolved phages, although measurements of small differences in absolute fitness may not be reliable [41].

### ΦX174 phenotypic adaptation to hosts blocking lysis: Failure to evolve optimally despite evolution

The slyD gene of *E. coli *is needed for lysis by wild-type ΦX174. However, in agreement with previous work [35], ΦX174 mutants that formed plaques on slyD-defective hosts were easily isolated. By definition, any mutant that lyses this host at all lyses earlier than the wild-type phage. We were interested in the lysis time of these mutant phages and of their descendants that were further adapted to the inhibitory host. A mix of two plaques was used to initiate an extended adaptation. After 35.5 hours of adaptation, the resultant phage (ΦX174_ΔslyD_) had a lysis interval of 11.2 min, similar to that of ΦX174_C _(11.4 min, Table 1). Not only are the lysis intervals indistinguishable between the evolved phages on their respective hosts, but the fitnesses are similar: 23.4 db/hr for ΦX174_C _versus 21.2 for ΦX174_ΔslyD _(*p *< 0.006). Again, the virus failed to reach its predicted optimum (5.5 min) by almost 6 min (*p *< 0.0052 by parametric test).

The mechanism of adaptation to slyD-deficient hosts is increased expression of the phage lysis gene E, which in a λ phage construct results in earlier lysis even in the presence of slyD [26]. By this criterion, a phage adapted to grow on the slyD host should lyse earlier when grown on normal hosts. Matching this expectation, ΦX174_ΔslyD _grown on normal hosts had a lysis interval of 8.9 min, shorter than the interval on slyD hosts (11.2 min, *p *< 0.025 by 2-tailed t-test).

On normal hosts, ΦX174_ΔslyD _lysis interval was also somewhat shorter than that of ΦX174_C_, although this difference was only marginally significant (Table 1, 8.9 min rather than 11.4 min, *p *< 0.1 by 2-tailed t-test). If this difference is real it would suggest that ΦX174_C _lyses later than predicted due to pleiotropic costs, rather than absolute constraint.

### Selecting for later lysis

If our model was wrong because it calculated an inaccurate optimum rather than an unattainable optimum, then a change in the growth conditions to select for later lysis should result in slower lysis after adaptation, because we know the virus can attain that phenotypic state. This can be tested by growing the phage at a sufficiently lower host density to favor longer lysis interval. The same starting population of phages used for ΦX174_ΔslyD _was grown on hosts at 2 × 10^6 ^cells/ml, which greatly lowered fitness. Even after 25.6 hr of adaptation, the final phage (ΦX174_ΔslyDlow_) had a much lower fitness (absolute growth rate) under these conditions than ΦX174_ΔslyD _had when grown at 1 × 10^8 ^cells/ml (Table 1, 10.6 rather than 20.4 db/hr). However, lysis interval evolved to a similar endpoint (10.0 min ± 0.11 min standard error for ΦX174_ΔslyDlow _as compared to 11.2 ± 0.54 min for ΦX174_ΔslyD _(*p *< 0.13 by 2-tailed t-test). Although the optimal lysis time for ΦX174_ΔslyDlow _(8.7 min) was considerably later than that of ΦX174_ΔslyD _(5.5 min), ΦX174_ΔslyDlow _still failed to attain its optimum (*p *< 0.0046 by parametric test).

### ST-1 phenotypic adaptation: Failure to evolve optimally despite evolution

**Figure 2:**
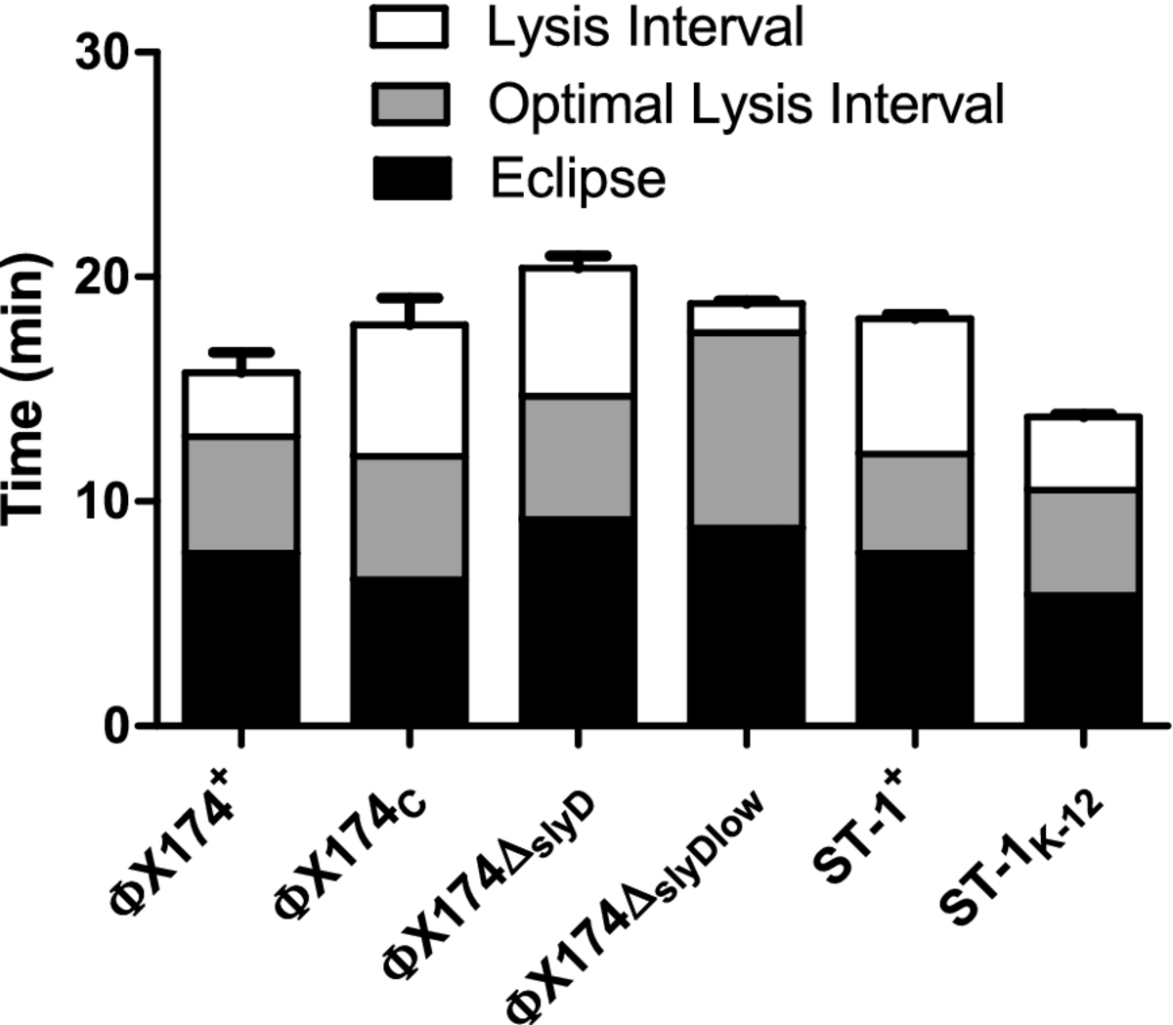
**Evolution of lysis interval**. Black bars represent eclipse time, gray bars represent optimal lysis interval, and gray + open bars represent the observed lysis interval. In all cases observed lysis interval is longer than the predicted optimum. Optimal lysis interval changes as a result of environmental changes or phenotypic evolution. Error bars for observed lysis interval show 1 standard error.

ST-1 initially had an eclipse time of 7.7 min and a lysis interval of 10.4 min. The phage adapted to laboratory conditions (ST-1_K-12_) had a significantly earlier eclipse time (*p *< 0.01) and shorter lysis intervals (*p *< 0.005), each by ~2 minutes, resulting in lysis approximately 4 min earlier than ST-1^+ ^(Table 1, Figure 2; see Additional file 1 for representative curves). Attachment rate was high throughout and did not change (Table 1), suggesting this adaptation was not driven by attachment evolution. Although the burst size estimate was somewhat smaller after adaptation, this difference was not significant (*p *< 0.17 by 2-tailed t-test).

The lysis interval of the evolved phage ST-1_K-12 _(8.0 min) did not reach the predicted optimum of 4.7 min (*p *< 0.004 by parametric test). This deviation has a predicted fitness cost of 4.4 db/hr. Thus, ST-1 lysis interval appears to have evolved qualitatively as expected, but did not reach the optimum despite selection to do so.

### Molecular evolution in both phages

Neither ΦX174_C _nor ST-1_K-12 _carried any coding or apparent regulatory changes in gene *E*, which generates the only protein known to play a role in ΦX174 lysis. Thus there is no obvious genetic cause for the decrease in lysis interval observed in ST-1. ΦX174_C _contained seven substitutions relative to ΦX174^+ ^(Table 2), two of which were not present in the preadapted founding population. ST-1_K-12 _acquired four substitutions, two during the initial adaptation to purely permissive hosts and two more in phase II when nonpermissive hosts were also present (Table 3).

**Table 2:** ΦX174 Genotypic Evolution

Nucleotide	Gene	Change	Gene Function	ΦX174_C_	ΦX174_C#2_	ΦX174_ΔslyD_	ΦX174_slyDlow_
756^† ‡^	*D*	T- > C F123L	External scaffolding protein	+			
756^† ‡^	*E*	T- > C R63R	Lysis	+			
873^† ‡^	*J*	G- > C G9A	DNA Packaging	+			
930^‡^	*J*	C- > T A28V	DNA Packaging	+			
1033^†^	*F*	G- > C A11P	Major coat protein	+			
1648^†^	*F*	C- > T P216S	Major coat protein	+			
3800	*H*	T- > C C868R	Minor spike protein	+			
4706^†^	*A*	G- > A S726S	DNA replication				
4706^†^	A*	G- > T208A	Nonessential				
1735	*F*	A- > G A245A	Major coat protein		+		
3339	*H*	G- > D137N	Minor spike protein		+		
575^§^	*D*	G- > A T62T	External scaffolding protein			+	
575^§^	*E*	G- > A R3H	Lysis			+	
576	*D*	C- > T L63L	External scaffolding protein			+	+
576	*E*	C- > T R3R	Lysis			+	+
624^§^	*D*	G- > T A79S	External scaffolding protein			+	+
624^§^	*E*	G- > T L19F	Lysis			+	+

All changes were identical between two sequenced ΦX174_C _isolates

^†^indicates mutation present in ΦX174 Af [42], one of the adapted lines used to start the adaptation

^‡^indicates mutation present in ΦX174_pif- _[43], the other adapted line with mutations available at the start of adaptation. All mutations in ΦX174_C _not present in ΦX174^+ ^were sequenced in ΦX174_pif-_

^£^Also silent in context of change at 575

^§^Mutations previously found to compensate for slyD hosts [40]

**Table 3:** ST-1 Genotypic evolution

Nucleotide	Gene	Change	Gene Function	End of phase I adaptation	ST-1_K-12_
807	*A*	T- > C V50A	DNA replication	+*	+
1387	*A*	T- > C T210T	DNA replication	-	+
3869	*F*	G- > T V210F	Major coat protein	-	+
5540	*H*	A- > C D149G	Minor spike	+	+

All mutations from ST-1_K-12 _were adaptive under conditions used for phase I of adaptation (R.H. Heineman, unpublished data)

*This mutation was only present in one of two sequenced isolates from this time point. The isolate with this additional change was used in all subsequent phenotypic assays

While many of the genetic changes of ΦX174_C _were present in the founding lines, two mutations were new. This shows that there was time for new substitutions to fix, and thus the potential for evolution closer to the optimum that did not rely on changes from the preadaptations. All 7 of the ΦX174_C _changes together only increased the fitness of the wild-type phage by 2.6 db/hr, suggesting that any single change moving lysis time near the optimum was not available.

In the two adaptations to slyD-deficient hosts, however, every change observed was in the lysis gene *E *(and overlapping gene *D*, Table 3). Two of these changes have been previously observed to restore lytic capacity on these hosts [26]. As the adaptations were started from the same population, no conclusion can be drawn from the similarity of genetic changes between the adaptations. However, they do demonstrate that gene *E *evolution changes lysis time under some conditions. If ΦX174_ΔslyD _does lyse faster on regular hosts than ΦX174C does, as seems possible (see previous section) the lack of these gene *E *changes in the other lines suggests that there is some cost to the changes that prevent them from spreading under normal conditions despite optimality predictions.

## Discussion

This study provides an experimental test of a quantitative optimality model in a phage system. Although optimality models are widely used to explain nature, there have been few attempts to actually evolve organisms to an optimum. Is it possible to predict phenotypic evolution using a simple model based largely on natural selection?

Optimality models may fail for a variety of reasons. An evolutionary constraint might prevent any adaptation at all from the starting, non-optimal state. For example, in a study of optimal foraging evolution, comparisons between ΦX174 and the related phage G4 demonstrated that selection would favor indiscriminate foraging under certain conditions, yet an absolute genetic constraint prevented adaptation of G4 to infect a new host [44]. However, another optimal foraging study using T7 showed that adaptation was possible for that phage [45]. Another form of constraint would be genes with pleiotropic effects on traits not included in the model. In Φ6, expansions in host range were often associated with decreased attachment to the original host [46] while in T7 a similar mechanism caused a failure of some optimal foraging predictions [45]. There is thus precedence for constraint on adaptation of simple phenotypes in phage genomes, but the extent of constraint is variable.

Phage lysis interval, and thus lysis time, is likely to be under strong and fluctuating selection in the wild, thus providing an ecologically important model system for the evolution of a life history trait. The lysis time optimality model is based on a trade-off between progeny number and generation time. It predicts how long a phage should wait after eclipse to lyse its host based on the growth rate of the phage, assuming that phage progeny accumulate linearly within the host after eclipse. The model has been previously tested in three phages: λ, T4 and T7. In all of those phages, lysis is thought to require a 2-component lysis mechanism; a lysin that destroys the cell wall and a holin that destroys the cell membrane allowing the lysin to access the cell wall. The holin triggers lysis with great precision in at least some phages [34].

The optimality model was tested in phage λ and T4 by competing lysis mutants with wild-type phages. In both cases, as predicted by the model, different environments selected for different mutants. [15,16,25]. In T7, quantitative optimality predictions were tested by allowing lysis to evolve without previously introduced genetic changes known to affect lysis. Adaptation had mixed success; selection for late lysis did not generate the optimal phenotype. Model failure was attributed to a violation of the model assumption that phage particles increase linearly inside the cell if the normal lysis mechanism is suppressed [17].

This prior work motivated the work here. How would the model fare in a one-component system such as ΦX174 and ST-1? ΦX174 satisfies the model assumption of linear accumulation [18]. In addition, mutations in the lysis gene (*E*) have been found that hasten lysis time under some conditions [26]. There was thus no a priori reason that the mean lysis interval for the population could not evolve to the optimum.

Under selection for a shorter lysis interval (according to the optimality model) ST-1 evolved earlier lysis by 4 min (although 2 min of this was caused by earlier eclipse), while ΦX174 did not; no changes in the ΦX174 lysis gene evolved in any adaptation to normal hosts. In none of the adaptations on either phage did lysis interval reach the predicted optimum. Quantitatively optimal evolution is not expected in a single step, because a large change is not likely to carry the phenotype directly to the optimum [47,48]. However, it is surprising how far from the optimum the phages in this study remained after adaptations involving a number of genetic changes.

While precise control of the phenotype appears difficult, it is clear that rapid evolution is possible in a different part of the phenotype space. When ΦX174^+ ^is grown on hosts lacking the cofactor slyD, it does not lyse detectably, yet the adapted phage attains an lysis interval on the slyD-defective hosts indistinguishable from that of a virus adapted to normal hosts. This is similar to the pattern seen in T7, which can recover from complete genetic ablation of lysis but cannot reach the predicted optimum [17,49]. Interestingly, despite the ability of ΦX174 to recover lysis on slyD-defective hosts, genetic details are relevant here as well; the coding mutations seen in our adaptations were the same as those previously found in another study [40], demonstrating a limited number of paths to maximal recovery and thus the possibility for constraint.

It could be that the phages would have reached the optimum if allowed to adapt for longer. However, repeated evolution of ΦX174 to a common phenotypic endpoint and the observation that phenotypic evolution was rapid over other areas of phenotype space suggest that this was not the primary limit on adaptation.

Instead, constraint on lysis interval, either absolute or due to pleiotropic effects on other phenotypes, seems the most likely explanation for failure to reach the predicted optimum. This may be caused by the overlap of gene *E *with gene *D*, which is highly conserved [28], or by the relatively crude ΦX174 lysis mechanism [26]. If evolution were possible but the quantitative optimality prediction itself was inaccurate, introducing major changes that would alter the optimum would lead to a different results, yet this had no effect.

A large part of the ST-1 lysis time adaptation was driven by changes in eclipse time. Phenotypes that are outside the scope of a model may frequently play a role in evolution. When optimality models are tested in nature, researchers often form conclusions after considering only the life history trait thought to be under selection. However, most life history traits, like lysis time, result from the interplay of a number of factors, which may mask the nature of adaptation.

This complexity is also evident on the genetic level. For example, ST-1 evolved to lyse closer to the optimum (shortening both its post-infection lysis time and its lysis interval) without changes in gene *E*, the only gene implicated in the widely accepted 1-component model for lysis in ΦX174-like phages [50]. Other genes (*A, F *and *H*) that did evolve in ST-1 may play some heretofore unsuspected role in mediating lysis. However, it is far more likely that, as previously observed in T7 phage, genes with no direct role in cell lysis affect lysis timing by altering timing of lysis gene expression or by indirect effects on other stages of the life cycle rather than lysis mechanisms per se [17,20,49]. Similar interactions may make optimality predictions in other organisms more difficult.

## Conclusion

Optimality models can be used in two ways: as a means of producing accurate phenotypic predictions or as null hypotheses. As null hypotheses, optimality models help us detect and explore violations of model assumptions. Here, for example, the quantitative optimality model was strictly required in order to identify constraint. However, it may be that even in well studied systems, genetic details make it difficult to use simple models to quantifiably predict the evolution of life history traits across taxa. The constraint we observed, the difference in model success between replicates from different species, and the evolution of traits outside the scope of the model are all consistent with this outlook.

## Methods

### Cell and phage lines

ΦX174 was grown on *E. coli *C cells. The ΦX174 adaptation was initiated with a wild-type ΦX174 allowed to recombine with two preadapted ΦX174 phages (by cross-streaking all phages to allow coinfection) in order to introduce genetic variation. One isolate, ΦX174 Af [42], had been adapted to *E. coli *C under the same conditions used here except that phage generations were restarted approximately every 35 minutes through addition of chloroform. The other, ΦX174_pif- _[43], was adapted without frequent chloroform treatment but on a different host, IJ1862 (*E. coli *C/F'128 *pifA15 lacZ*Δ*M15*Tn*10*). This recombinant population was used deliberately to provide potentially adaptive substitutions to the wild-type phage and thus maximize the potential evolution. The source of all changes in the resultant evolved line was determined by sequencing. ΦX174_ΔslyD _and ΦX174_ΔslyDlow _were adapted to slyD-defective host CCX1 (*E. coli *C *slyD zhd*::Tn10)[39].

ST-1 phage [51,52] was adapted on *E. coli *K-12 strain BW25113 (*rrn*B3 Δ*lacZ*4787 *hsd*R514 Δ(*ara*BAD)567 Δ(*rha*BAD)568 *rph*-1) with an additional deletion of *lacY*, part of the Keio collection [53]. Another Keio collection mutant (Δ*rep*), identical except that it instead lacked the *rep *helicase protein, made up 95% of the cells in the second phase of ST-1 adaptation. The Δ*rep *host was nonpermissive, as ST-1 infects it but does not produce viable phage offspring [54], and thus its presence lowered overall fitness.

### Adaptation

Adaptation protocols were similar to that described in other work [17] with some modifications. Specifically, 10 ml of LB media (10 g NaCl, 10 g Bacto tryptone, 5 g Bacto yeast extract), supplemented with 5 mM CaCl_2 _to improve adsorption of both Φ174 and ST-1 to their hosts, was pre-warmed to 37°C in an orbital water bath (200 rpm). Cells from recently thawed 20% glycerol-LB stocks stored at -80°C were then added and allowed to grow for an hour such that the cell population was 1-2 × 10^8 ^cells/ml (1-2 × 10^6 ^cells/ml for ΦX174_ΔslyDlow_) and in exponential phase when phages were added.

Phages were incubated in this cell culture for only 20-30 min, minimizing changes in host density over time, which would confound the optimality model predictions. Following incubation, an aliquot of the infected culture - which included both free phage and infected cells - was transferred to a new flask in which cells were at the requisite density (having grown one hour). The amount transferred was such that roughly 10^5 ^phages total (no less than 10^4 ^and no more than 10^7^) were added to the next flask. In addition, at the end of each flask, a sample of the completed flask was chloroform-treated and stored, preserving free phage and killing remaining hosts. The chloroformed sample from the final flask of the day was then used as the starting population for the first flask of the next day.

ΦX174 was adapted for 34 hours (excluding the pre-adaptation time of the starting populations) to yield ΦX174_C_, with a second adaptation of ΦX174^+ ^over 35 hours to get ΦX174_C#2_. Both adaptations to slyD-defective hosts started from a 1:1 mixture of two mutant plaques. ΦX174_ΔslyD _was adapted for 35.5 hr, while ΦX174_ΔslyDlow _was adapted for 26.6 hr. ST-1 was adapted for 26.3 hours on purely permissive hosts in phase I of adaptation, then on a mix of 95% nonpermissive and 5% permissive hosts for 41.5 hours in phase II of adaptation (resulting in ST-1_K-12_). The latter conditions did not change the direction of selection on lysis time, but merely facilitated longer, more convenient passages. The nonpermissive hosts reduced phage growth rate and thus prolonged the time before phage density exceeded cell density, so transfers could be performed less often. This lower fitness led to a slightly later optimal lysis time (which ST-1_K-12 _still did not attain). The mutagen N-methyl-N'-nitro-N-nitrosoguanidine was added at a concentration of 0.5 ug/ul to one flask at 17.5 hr of the ST-1 adaptation to promote adaptation by generating new mutations.

### Fitness assay

Viral fitness was measured under conditions essentially identical to those used for adaptation. The rate of increase was determined by growth at low phage/cell ratios (not exceeding 0.1 by the end of transfer) across five consecutive transfers, using phage titers measured from the end of the second flask to the end of the fifth [see 17 for details]. Fitness is reported as doublings per hour, calculated as [log _2_(N_t_/N_0_)]/t, where N_t _is the total number of phage in the flask at time t hours, corrected for dilutions over multiple transfers. The intrinsic rate of increase per minute, *r*, is simply ln(2)/60 times the fitness in db/hr, but db/hr is easier to comprehend.

### Eclipse time, lysis interval and burst size

To measure eclipse, *E*, 5 × 10^7 ^phages were added to 10 ml of LB with 5 mM CaCl_2 _and cells previously grown to 10^8^/ml in an orbital water bath, then incubated for three minutes. Cultures were diluted 10^3 ^fold in a new (preheated) flask to halt further adsorption. Samples were removed at a number of time points and diluted 10-fold in HFB [55] containing 2 mg/ml lysozyme saturated with chloroform, which lyses cells infected with ΦX174-like phages [56]. Eclipse time was estimated by plating these samples, then fitting the resultant curve to an empirical least squares model [17].

A similar protocol was used to determine lysis interval and burst size of phage infections. Cultures were diluted 10^4^-fold and 10^5^-fold to new flasks after 3 min. The total number of free phage plus infected cells was estimated by direct plating of appropriate dilutions; plating after chloroform treatment but prior to 7.5 min (before progeny production) gave the number of free phage. This allowed calculation of infected cell density. Burst size was calculated by comparing this number with the final phage count after phage production had reached a plateau (in chloroform-treated samples, ~30 min after infection).

The data from direct plating at a number of time points allowed determination of lysis time and variance of lysis by an empirical least squares model [17]. The asynchrony of lysis is reported as the standard deviation (σ) of the time infected cells took to lyse, and is larger when individual cells in the population lyse at very different times due to phenotypic noise.

### Attachment assays

Plaques were resuspended in LB within one day of plating and used for attachment assays within 3 days to ensure freshness. Between 10^5 ^- 10^6 ^phages were added to cells grown to 10^8 ^cells/ml at 37°C (as described above). After 5 min, part of the sample was spun down to remove attached phages. Spun and unspun cultures were plated to calculate free (*N_free_*) and total (*N_total_*) phage densities. Adsorption rate *α *was calculated from *N_free _*= *N*_*total *_× *e*^-5*Cα*^, where *C *is the cell density in cells/ml.

### Sequencing

Phage genome sequences used dideoxy chain termination reactions with ABI Big Dye Terminator and were analyzed with an ABI3100 automated sequencer. Sequence files were checked and compared with DNA Star Lasergene Seqman software (version 5.05). All sequencing was from polymerase chain reaction products, except the wild-type ST-1, which was sequenced from genomic DNA. The entire phage genomes from the end of ST-1 phase I adaptation, ST-1_K-12_, and ΦX174_C _were sequenced from two isolates apiece, and one of the sequenced clones from each was selected randomly for phenotypic assays. ΦX174_pif-_, one of the initial phages for the ΦX174 adaptation, was sequenced over regions that differed between the ΦX174^+ ^phage and ΦX174_C _in order to determine which changes arose during the final adaptation. All ΦX174 DNA nucleotide changes are reported using the reference ΦX174 sequence [GenBank: NC_001422].

### ST-1 genome

The full genome of ST-1 was sequenced and is reported as part of this study [GenBank: GQ149088]; homologues to all ΦX174 genes were found. ST-1's genome is most similar to the ΦK and α3 genomes, which are ΦX174-like but members of a distinct clade [28]. The genome includes a region encoding a number of putative genes that are homologous to some other ΦX174-like phages, such as α3 and WA13, but not ΦX174 itself [28].

### Optimality model

The simple optimality model for lysis presented in the background can be extended to account for the effects of lysis time variance, which is important for ΦX174 and may play a role in other phages as well [57]. If

(2) $$\hat{L}-E=\hat{X}$$

(where $\hat{L}$ is the lysis time at equilibrium and *E *the invariant eclipse time), and the lysis interval (*L *- *E*) is assumed to vary in a gamma distribution with variance *σ*^2 ^between individual infections, the following equation

(3) $$1+\hat{r}\hat{X}+2\hat{r}\frac{\sigma^{2}}{\hat{X}}-2\hat{X}\left(\hat{r}+\frac{\hat{X}}{\sigma^{2}}\right)\text{ln}\left(1+\hat{r}\frac{\sigma^{2}}{\hat{X}}\right)=0$$

is obtained [17]. This can be solved numerically for $\hat{X}$. This equation is used throughout this paper to predict optima.

Another equation allows calculation of expected phage fitness (*r*), which is useful when trying to determine how much higher the fitness would be if lysis interval changed.

$$r=\text{R}ce^{-rE}\frac{\mu }{{\left(1+r\frac{\sigma^{2}}{\mu }\right)}^{\left(\frac{\mu^{2}}{\sigma^{2}}\right)+1}}-c$$

when lysis interval has a gamma distribution with mean *μ *and variance σ^2 ^[17]. R is the constant rate at which phage accumulate within a cell between eclipse and lysis, *c *is the product of cell density and adsorption.

### Estimating fitness benefit of optimal lysis

The expected fitness of phages was calculated based on empirical parameters of eclipse time, lysis time, lysis variance, intracellular growth rate, and adsorption by solving equation (4) numerically. This parameter-based fitness estimation, which usually differed from direct measurements of fitness, was used to approximate the fitness effect of setting lysis time equal to the optimum.

### Statistical analysis

To test whether *L *- *E *- *X *= 0 (that is, whether the lysis time was at an optimum in an environment), we used empirical data to simulate a normal distribution for eclipse time, post-infection lysis time, lysis variance, and *r *(growth rate), parameterized with the means and variances calculated from the data. Numbers were randomly drawn from these distributions, with all numbers less than zero rejected as biologically implausible. Lysis variance and *r *from the distributions were used to solve for *X *by equation (3). This was repeated 5000 times, recording the proportion of times *L *- *E *- *X *had the opposite sign from the mean deviation from the optimum. The result multiplied by two is the 2-tailed probability that the means were identical. The significance of all other differences was determined by *t*-tests assuming unequal variances [58-63].

## Competing interests

The authors declare that they have no competing interests.

## Authors' contributions

LC performed the adaptations, sequencing, the majority of the phenotypic assays, and helped draft the manuscript. RH performed calculations of predicted optima, phage release assays with preadsorption, adsorption assays, and drafted the manuscript. All authors read and approved the final manuscript.

## Supplementary Material

Additional file 1 **Release curves for ST-1^+ ^(solid line) and ST-1_K-12 _(dotted line)**. (A) Samples treated with lysis solution containing 2 mg/ml lysozyme and chloroform, used to estimate eclipse. (B) Untreated samples, used to estimate lysis and lysis interval. Click here for file
