# Supplementary figures and images for: A common, non-optimal phenotypic endpoint in experimental adaptations of bacteriophage lysis time
Source: BMC Evol Biol. 2012 Mar 19;12:37. doi: 10.1186/1471-2148-12-37 (PMC3324380; doi:10.1186/1471-2148-12-37)

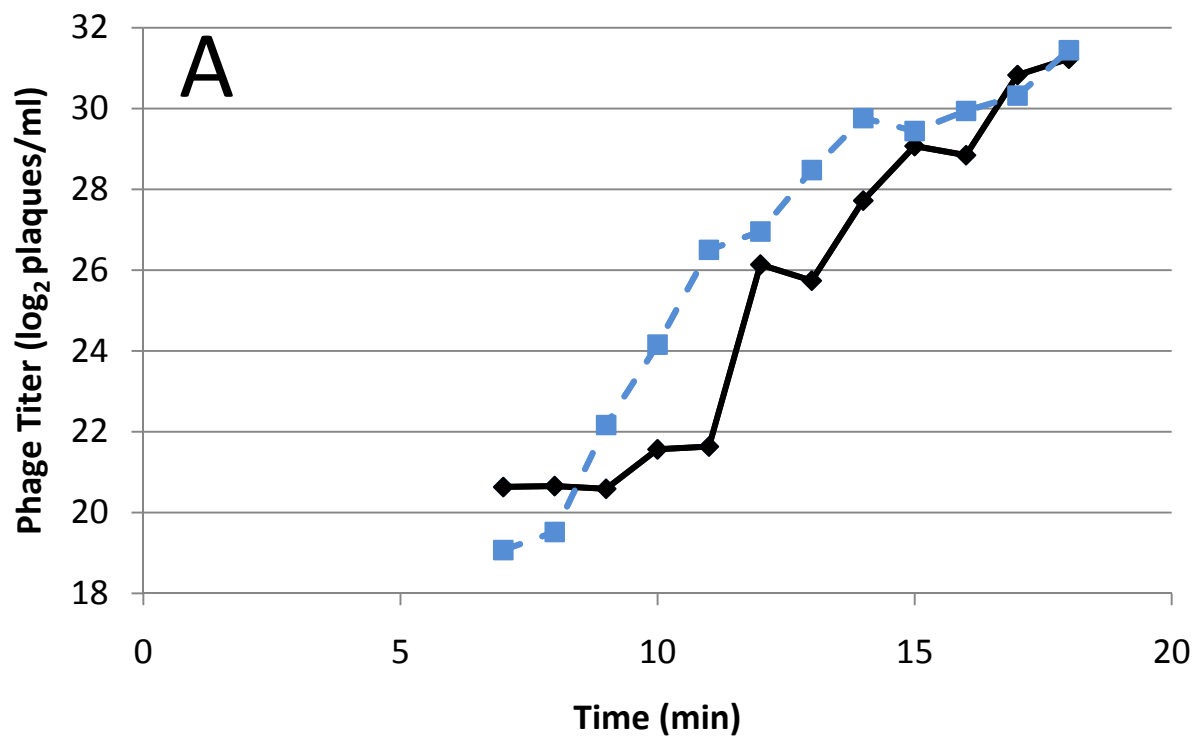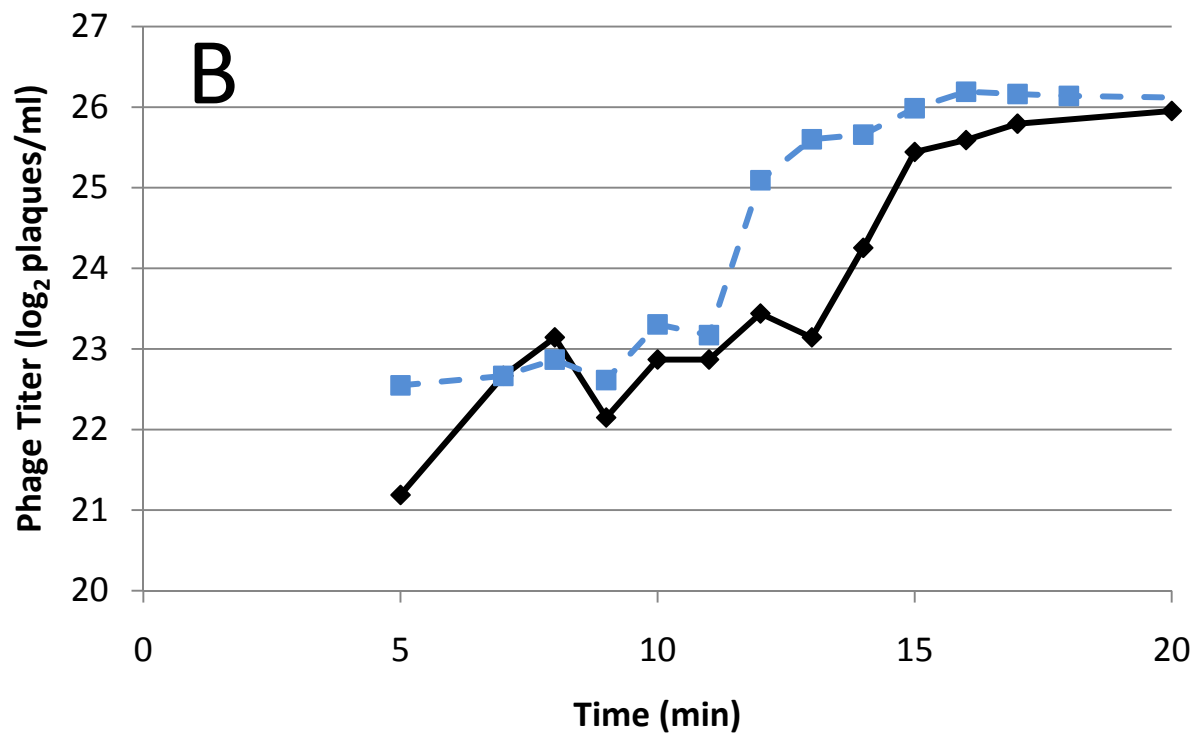

Supplement: Additional file 1 — Release curves for ST-1+ (solid line) and ST-1K-12 (dotted line). (A) Samples treated with lysis solution containing 2 mg/ml lysozyme and chloroform, used to estimate eclipse. (B) Untreated samples, used to estimate lysis and lysis interval. [file 1471-2148-12-37-S1.PDF]
